# Supplementary material for: PRC2 specifies ectoderm lineages and maintains pluripotency in primed but not naïve ESCs
Source: Nat Commun. 2017 Sep 22;8:672. doi: 10.1038/s41467-017-00668-4 (PMC5610324; doi:10.1038/s41467-017-00668-4)
Supplement: Supplementary file 1 — Supplementary Information [file 41467_2017_668_MOESM1_ESM.pdf]

Description of Supplementary Files

File Name: Peer Review File

File Name: Supplementary Information
Description: Supplementary Figures, Supplementary Tables.

Supplementary Figure 1

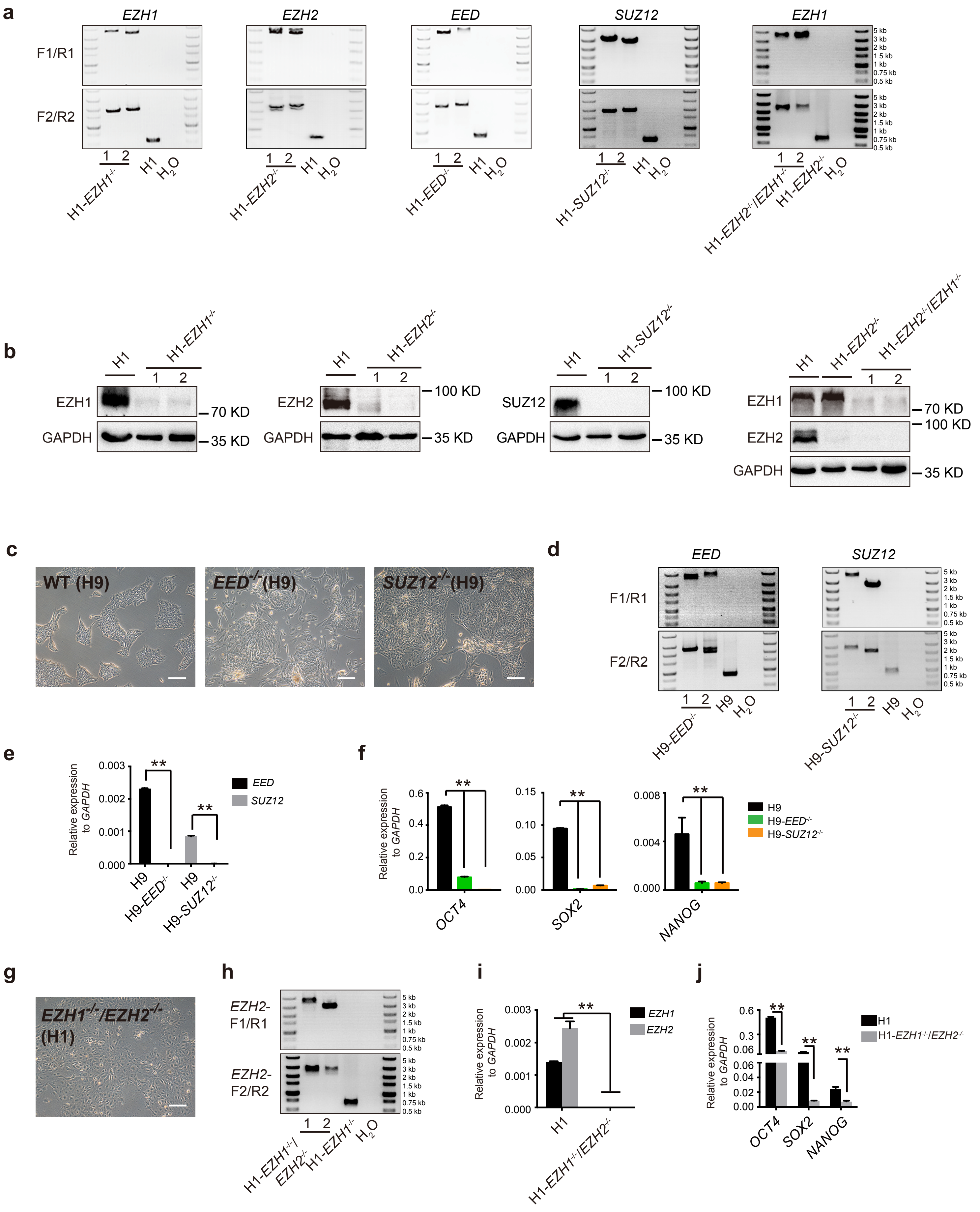

Supplementary Figure 1 Deletion of polycomb repressive complex 2 in human embryonic stem cells

(**a**) PCR identification of H1 hESCs with targeted deletion of each PRC2 component. Wild type H1 hESCs serve as a negative control. kb: Kilobase. (**b**) Western blot analysis of targeted PRC2 proteins in each knockout hESC line. GAPDH as the loading control. KD: Kilodaltons. (**c**) Morphology of H9 hESCs with the deletion of *EED* and *SUZ12*. Scale bar, 200 μm. (**d**) Representative PCR analysis of deletion of *EED* and *SUZ12* in H9 ESCs, respectively. Wild type H9 hESCs serve as a negative control. kb: Kilobase. (**e, f**) qRT-PCR analysis on the expression level of targeted genes and pluripotent genes in *EED*$^{-/-}$ and *SUZ12*$^{-/-}$ H9 ESCs. Significance level were determined using unpaired two-tailed Student' s t-tests. **, *P* <0.01. Data represent mean ± SD from three biological repeats. (**g**) Morphology of H1-*EZH1*$^{-/-}$ hESCs with targeting deletion of its homolog *EZH2*. Scale bar, 200 μm. (**h**) Representative PCR analysis of deletion of EZH2 in H1-*EZH1*$^{-/-}$/*EZH2*$^{-/-}$ ESCs. kb: Kilobase. (**i, j**) qRT-PCR analysis on the expression level of *EZH1*, *EZH2* and pluripotent genes in H1-*EZH1*$^{-/-}$/*EZH2*$^{-/-}$ ESCs. Significance level were determined using unpaired two-tailed Student' s t-tests. **, *P* <0.01. Data represent mean ± SD from three biological repeats.

Supplementary Figure 2

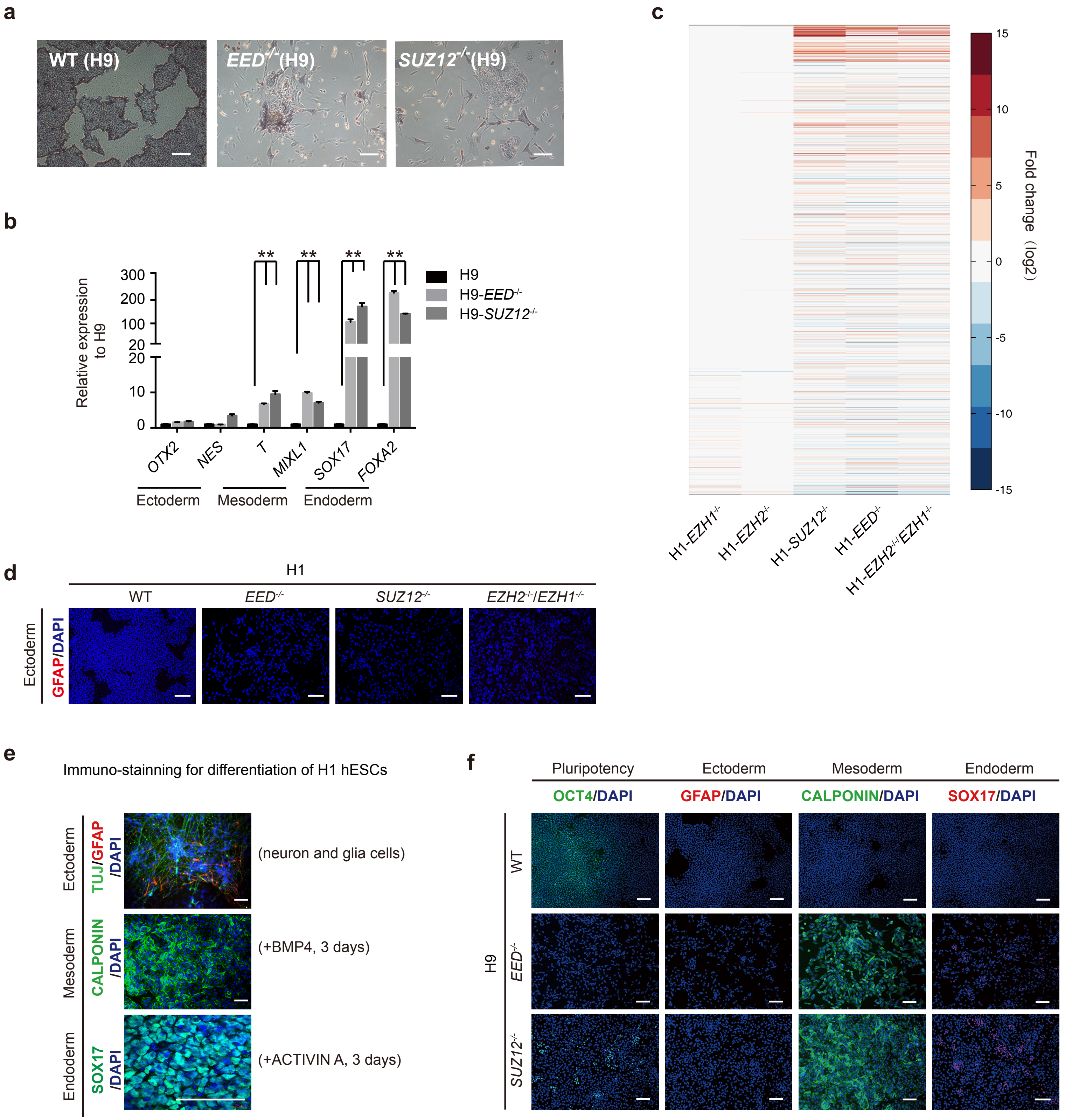

Supplementary Figure 2 PRC2$^{-/-}$ hESCs exhibit spontaneous differentiation to meso-endoderm fate

(**a**) Alkaline phosphatase (ALP) activity staining in *EED*$^{-/-}$ and *SUZ12*$^{-/-}$ H9 hESCs. Scale bar, 200 µm. (**b**) qRT-PCR analysis on the expression level of lineage-specific genes in *EED*$^{-/-}$ and *SUZ12*$^{-/-}$ H9 hESCs. Significance level were determined using unpaired two-tailed Student' s t-tests. **, *P* <0.01. Data represent mean ± SD from three independent repeats. (**c**) Heatmap analysis for differentially expressed genes in H1 hESCs with deletion of each component of PRC2 compared to H1 hESCs, respectively. (**d**) Immunostaining on GFAP (ectoderm) in *EED*$^{-/-}$ H1, *SUZ12*$^{-/-}$ H1 and *EZH2*$^{-/-}$/ *EZH1*$^{-/-}$ H1 hESCs. Scale bar, 100 µm. (**e**) Immunostaining on GFAP (ectoderm), CALPONIN (mesoderm), SOX17 (endoderm) in differentiated H1 hESCs. Scale bar, 100 µm. (**f**) Immunostaining on pluripotent and lineage markers, OCT4 (pluripotency), GFAP (ectoderm), CALPONIN (mesoderm), SOX17 (endoderm) in *EED*$^{-/-}$ and *SUZ12*$^{-/-}$ H9 hESCs. Scale bar, 100 µm.

Supplementary Figure 3

a

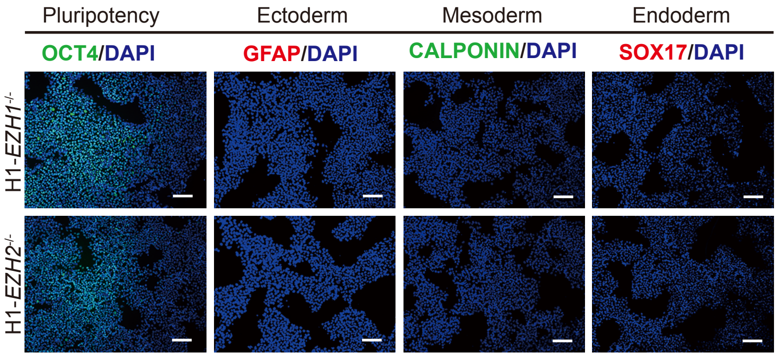

b

Cell line	H1	H1-*EZH1*$^{-/-}$	H1-*EZH2*$^{-/-}$
Number of teratomas	3	3	3
Sections of each teratoma	3	3	3
Number of ectoderm	35/45/25	0/0/0	0/0/1
Number of mesoderm	8/15/27	25/11/23	38/30/31
Number of endoderm	10/6/37	30/29/30	36/34/40

Supplementary Figure 3 *EZH1* and *EZH2* specify early neural ectoderm fate

(**a**) Immunostaining on pluripotent and lineage markers, OCT4 (pluripotency), GFAP (ectoderm), CALPONIN (mesoderm), SOX17 (endoderm) in *EZH1*$^{-/-}$ and *EZH2*$^{-/-}$ H1 hESCs. Scale bar, 100 μm. (**b**) Statistical result of teratomas formed by the indicated hESC cell lines.

Supplementary Figure 4

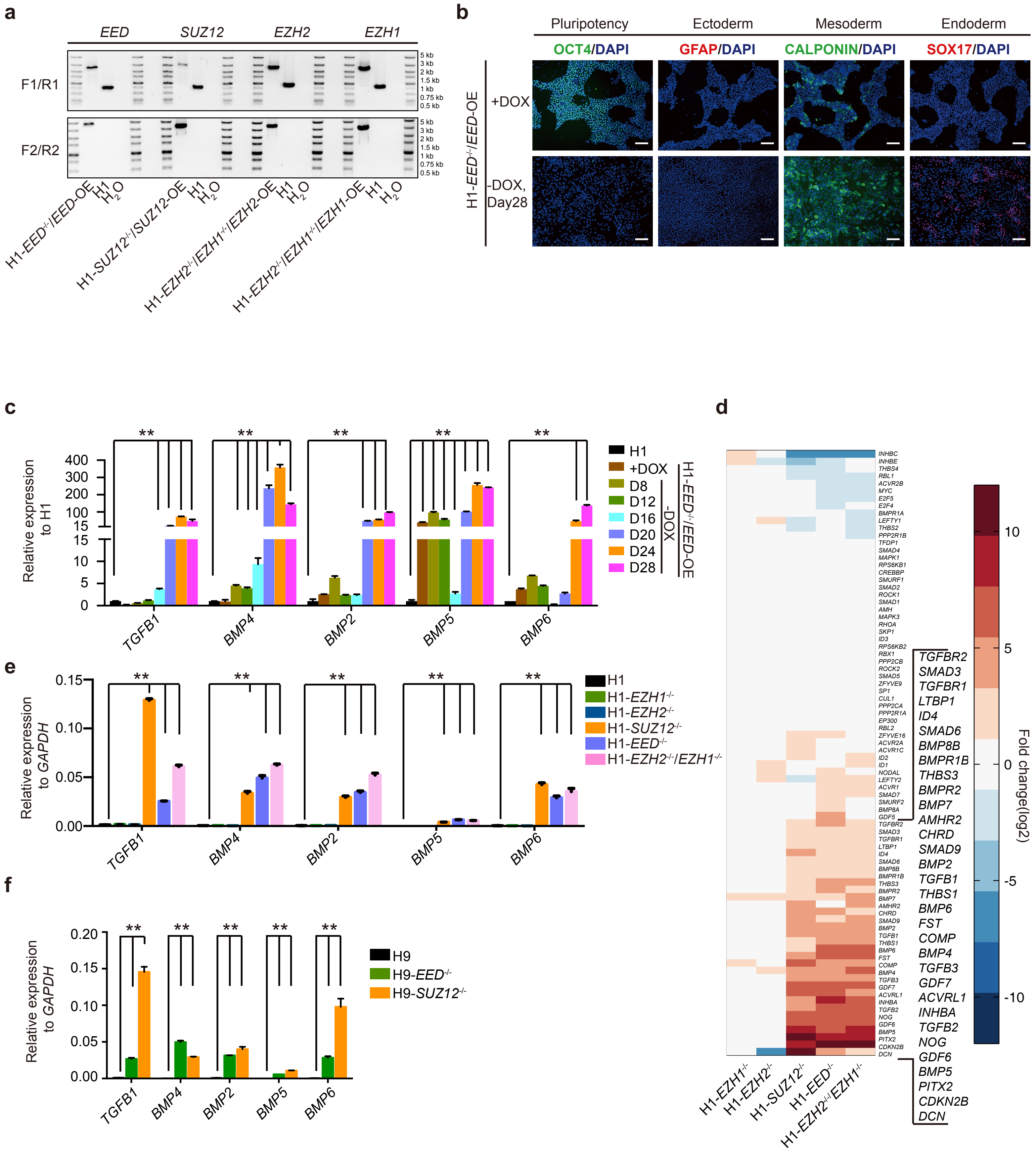

Supplementary Figure 4 PRC2 deletion preferentially induces BMP signaling in hESCs

(**a**) PCR identification of H1 hESCs with the inducible deletion of each PRC2 component. kb: Kilobase. (**b**) Immunostaining on pluripotent and lineage markers in H1-*EED*$^{-/-}$/*EED*-OE with and without DOX treatment at day 28. Scale bar, 100 μm. (**c**) Representative gene expression analysis of TGF-β/BMP signaling factors in H1-*EED*$^{-/-}$/*EED*-OE at different time points under DOX withdrawal. Significance level were determined using unpaired two-tailed Student' s t-tests. **, *P* <0.01. Data represent mean ± SD from three independent repeats. (**d**) Heatmap on TGF-β/BMP member genes in H1 hESCs with deletion of each component of PRC2 compared to H1 hESCs, respectively. (**e** and **f**) qPCR verification of TGF-β/BMP related gene expression in PRC2-gene targeting knockout H1 and H9 ESCs. Significance level were determined using unpaired two-tailed Student' s t-tests. **, *P* <0.01. Data represent mean ± SD from three independent repeats.

Supplementary Figure 5

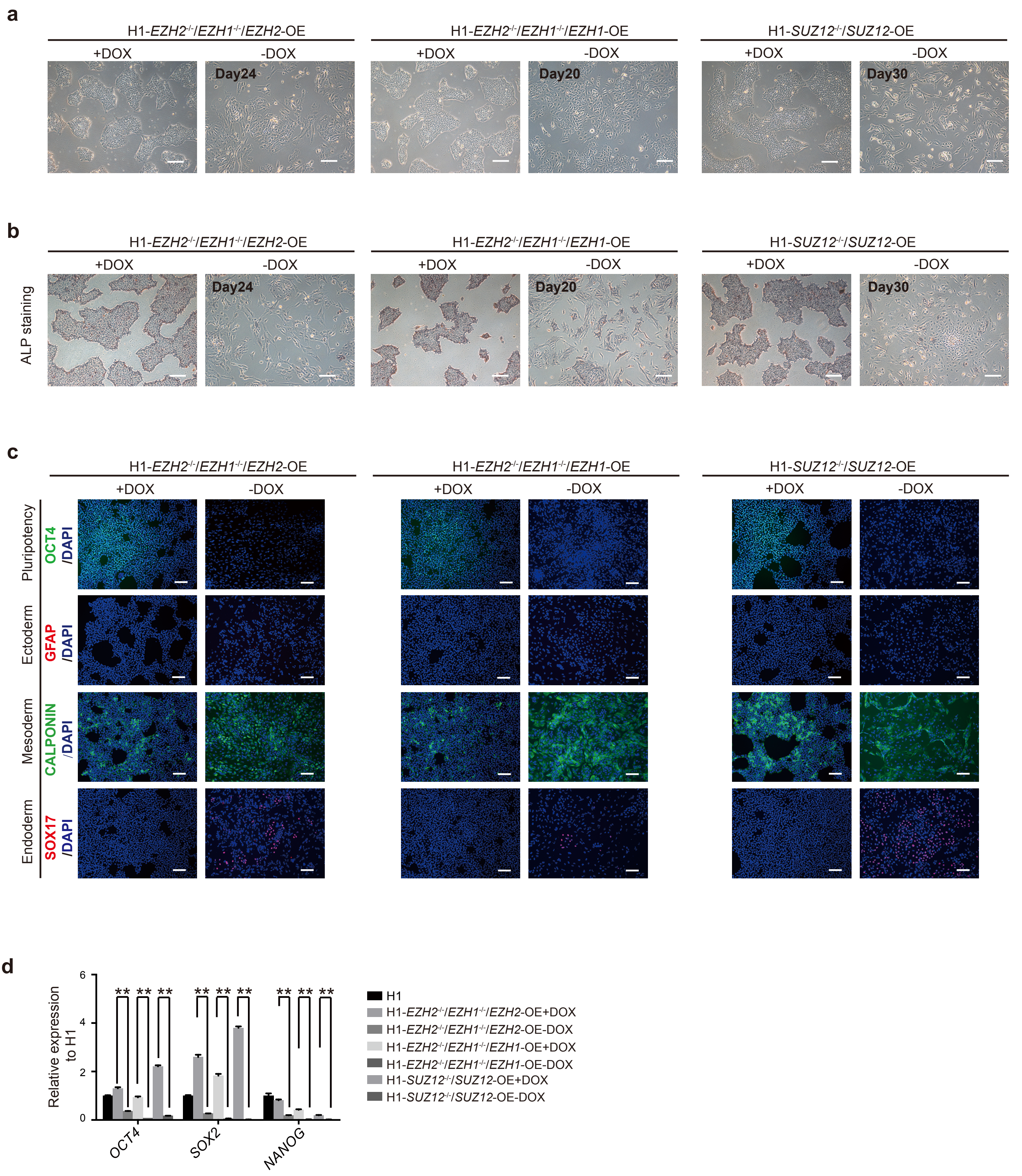

Supplementary Figure 5 Inducible knockout system in other components of PRC2

(**a** and **b**) Morphology and alkaline phosphatase (ALP) activity staining in H1-*EZH2*$^{-/-}$/*EZH1*$^{-/-}$/*EZH2*-OE, H1-*EZH2*$^{-/-}$/*EZH1*$^{-/-}$/*EZH1*-OE and H1-*SUZ12*$^{-/-}$/*SUZ12*-OE with and without DOX treatment. Scale bar, 200 µm. (**c**) Immunostaining on pluripotent and lineage markers in H1-*EZH2*$^{-/-}$/*EZH1*$^{-/-}$/*EZH2*-OE, H1-*EZH2*$^{-/-}$/*EZH1*$^{-/-}$/*EZH1*-OE and H1-*SUZ12*$^{-/-}$/*SUZ12*-OE with and without DOX treatment. Scale bar, 100 µm. (**d**) Representative gene expression analysis of pluripotent and lineage-specific genes in H1-*EZH2*$^{-/-}$/*EZH1*$^{-/-}$/*EZH2*-OE, H1-*EZH2*$^{-/-}$/*EZH1*$^{-/-}$/*EZH1*-OE and H1-*SUZ12*$^{-/-}$/*SUZ12*-OE with and without DOX treatment. Significance level were determined using unpaired two-tailed Student' s t-tests. **, P <0.01. Data represent mean ± SD from three independent repeats.

Supplementary Figure 6

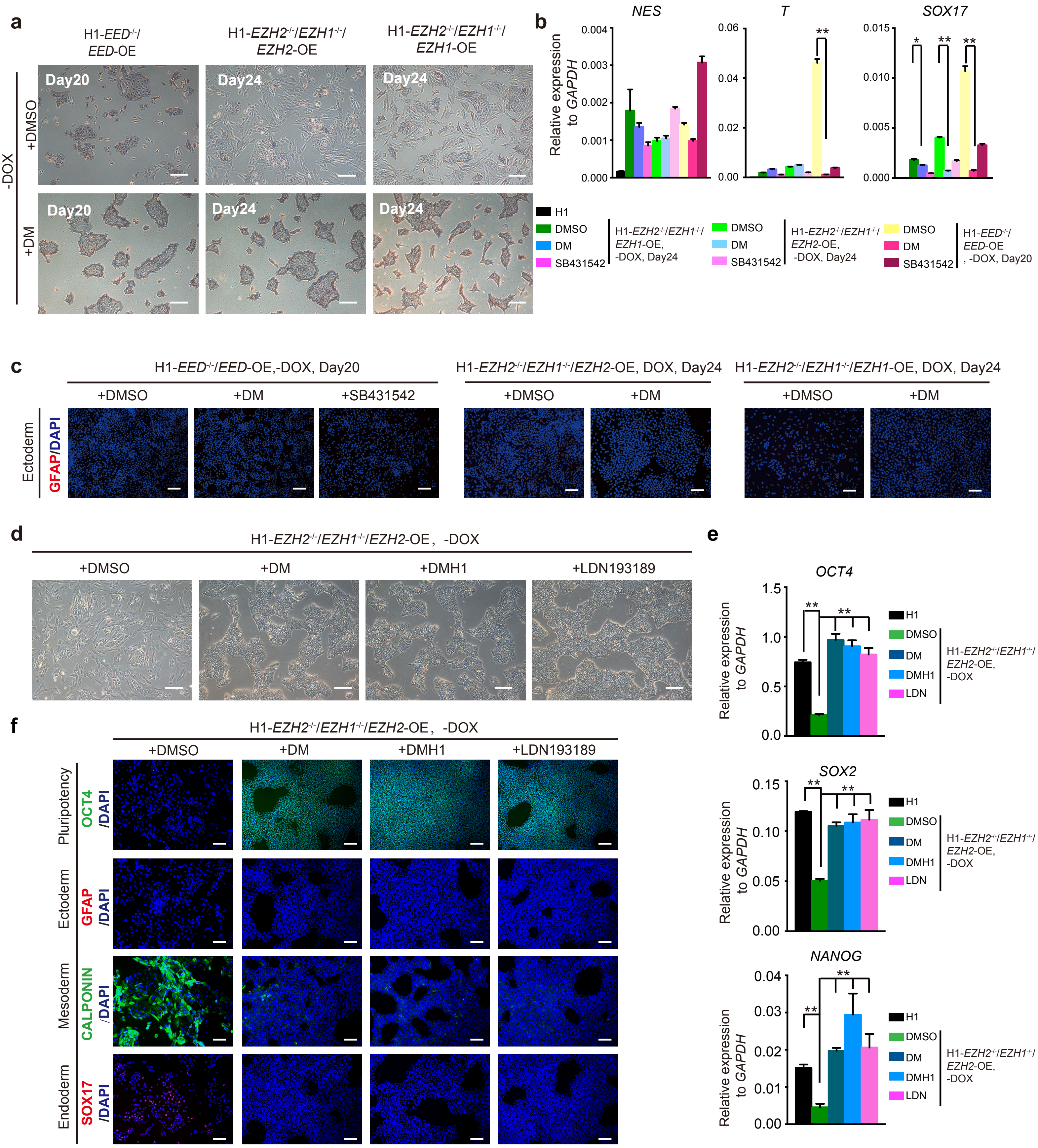

Supplementary Figure 6 Inhibition of BMPs signaling rescues PRC2 deficiency in hESCs

(**a**) ALP staining in H1-*EZH2*$^{-/-}$/*EZH1*$^{-/-}$/*EZH2*-OE, H1-*EZH2*$^{-/-}$/*EZH1*$^{-/-}$/*EZH1*-OE and H1-*EED*$^{-/-}$/EED-OE cultured in DOX withdrawal or plus DM. Scale bar, 200 µm. (**b**) Gene expression of lineage-specific genes in above those cell lines in DOX withdrawal, plus DM or SB431542. Significance level were determined using unpaired two-tailed Student' s t-tests. **, *P* <0.01. Data represent mean ± SD from three independent repeats. (**c**) Immunostaining on GFAP (Ectoderm) in above those cell lines in DOX withdrawal, plus DM or SB431542. Scale bar, 100 µm. (**d**) Morphology of H1-*EZH2*$^{-/-}$/*EZH1*$^{-/-}$/*EZH2*-OE hESCs cultured in defined medium with other BMPs inhibitors DMH1, LDN193189. Scale bar, 200 µm. (**e**) Expression of pluripotent genes OCT4, SOX2 and NANOG in the indicated hESCs with different treatments. Significance level were determined using unpaired two-tailed Student' s t-tests. **, *P* <0.01. Data represent mean ± SD from three independent repeats. (**f**) Immunostaining on the pluripotent and lineage markers, OCT4 (pluripotency), GFAP (ectoderm), CALPONIN (mesoderm), SOX17 (endoderm) in the indicated hESCs with different treatments. Scale bar, 100 µm.

Supplementary Figure 7

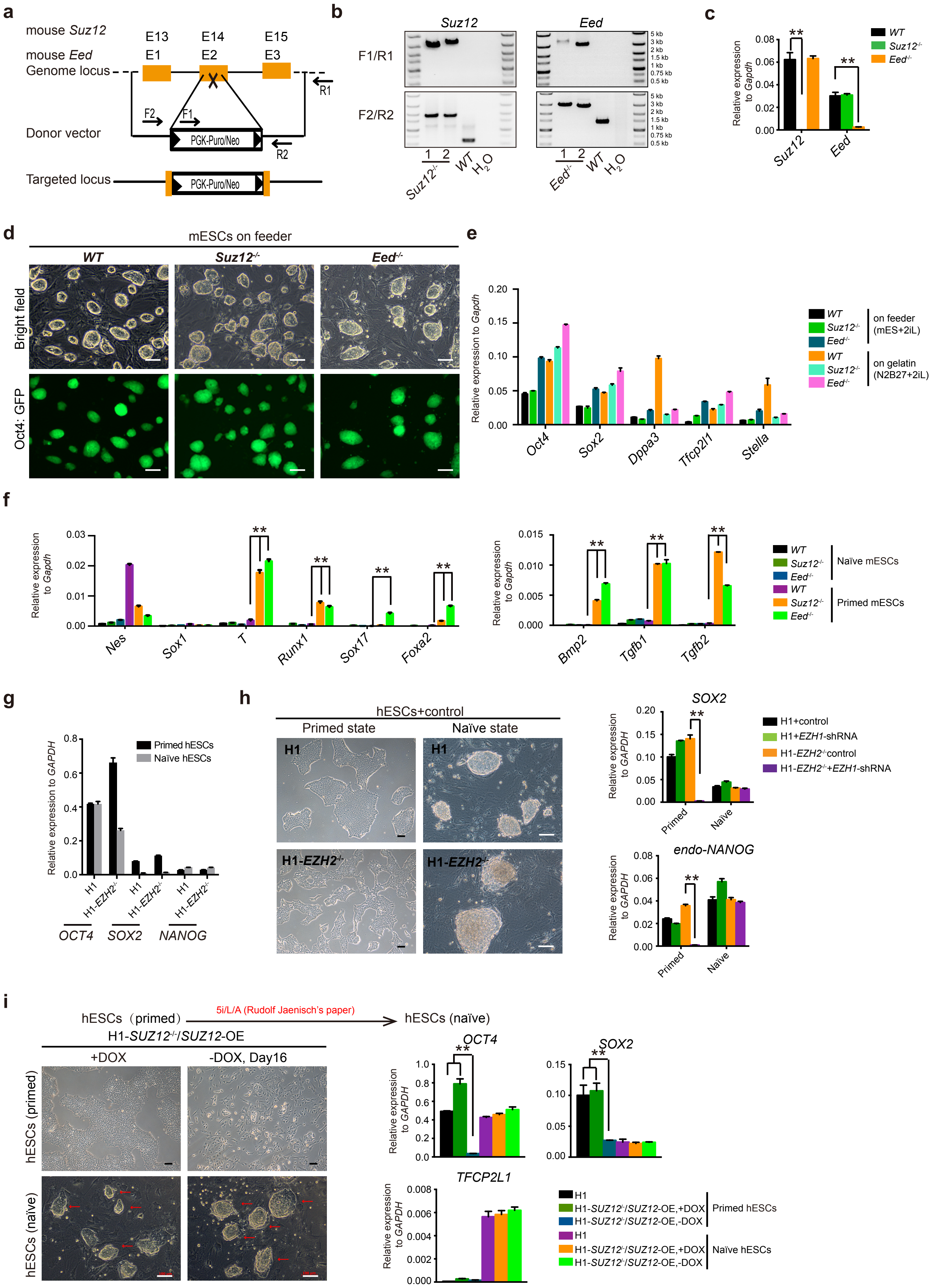

Supplementary Figure 7 PRC2 is required for pluripotency in primed not naïve state

(**a**) Overview of gene targeting strategy towards *Suz12* or *Eed* in mouse ESCs. (**b-c**) PCR and qRT-PCR identification of OG2 mESCs with targeted deletion of *Suz12* and *Eed.* kb: Kilobase. Significance level were determined using unpaired two-tailed Student' s t-tests. **, *P* <0.01. Data represent mean ± SD from three independent repeats. (**d**) Morphology of naïve mESCs with GFP expression controlled by Oct4 promoter (Oct4 : GFP) with *Suz12* and *Eed* deletion maintained on MEF. Scale bar, 100 μm. (**e**) qRT-PCR for expressions of the pluripotent and naïve marker genes. (**f**) qRT-PCR analysis on gene expression of lineage-specific genes and TGF-β/BMP signaling factors in primed and naïve mESCs with deletion of *Suz12* and *Eed*. Significance level were determined using unpaired two-tailed Student' s t-tests. **, *P* <0.01. Data represent mean ± SD from three independent repeats. (**g**) qRT-PCR analysis on the expression level of pluripotent genes in WT and H1-*EZH2*$^{-/-}$ hESCs in primed and naïve state following Austin Smith' s paper. (**h**) Morphology and qRT-PCR for expressions of the pluripotent genes in WT and H1-*EZH2*$^{-/-}$ hESCs with control of *EZH1*-shRNA at primed and naïve state. Scale bar, 100 μm. Significance level were determined using unpaired two-tailed Student' s t-tests. **, *P* <0.01. Data represent mean ± SD from three independent repeats. (**i**) Morphology and qRT-PCR for expressions of the pluripotent genes in WT and H1-*SUZ12*$^{-/-}$/ *SUZ12*-OE hESCs with and without DOX in primed and naïve state. Scale bar, 100 μm. Significance level were determined using unpaired two-tailed Student' s t-tests. **, *P* <0.01. Data represent mean ± SD from three independent repeats.

Supplementary Figure 8

a

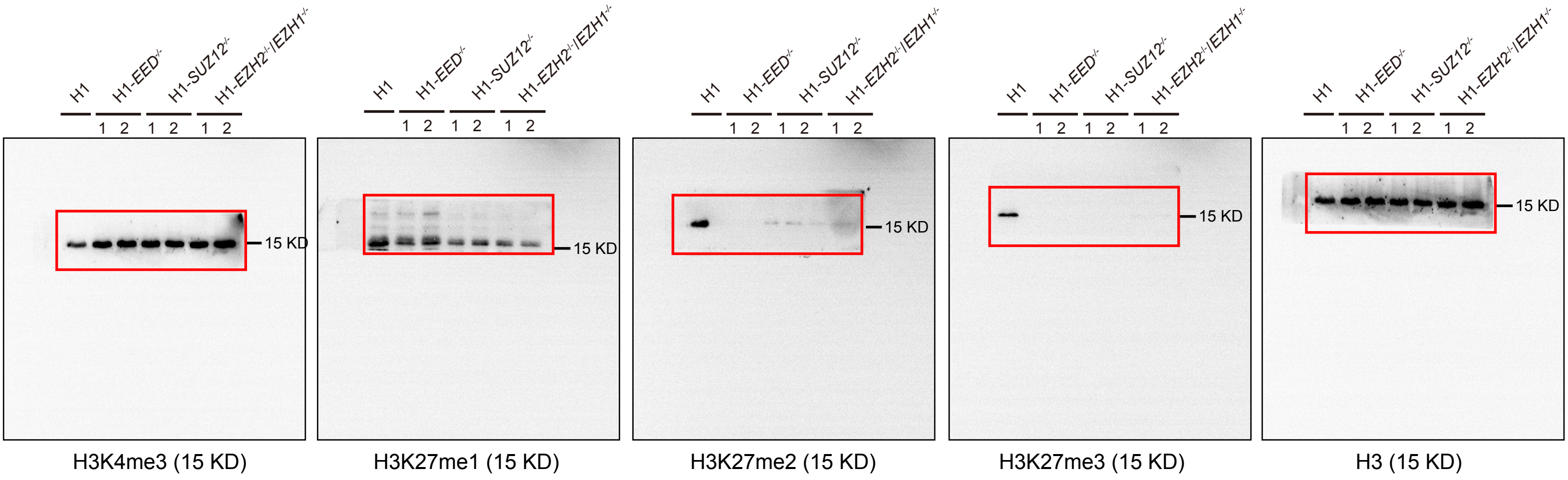

b

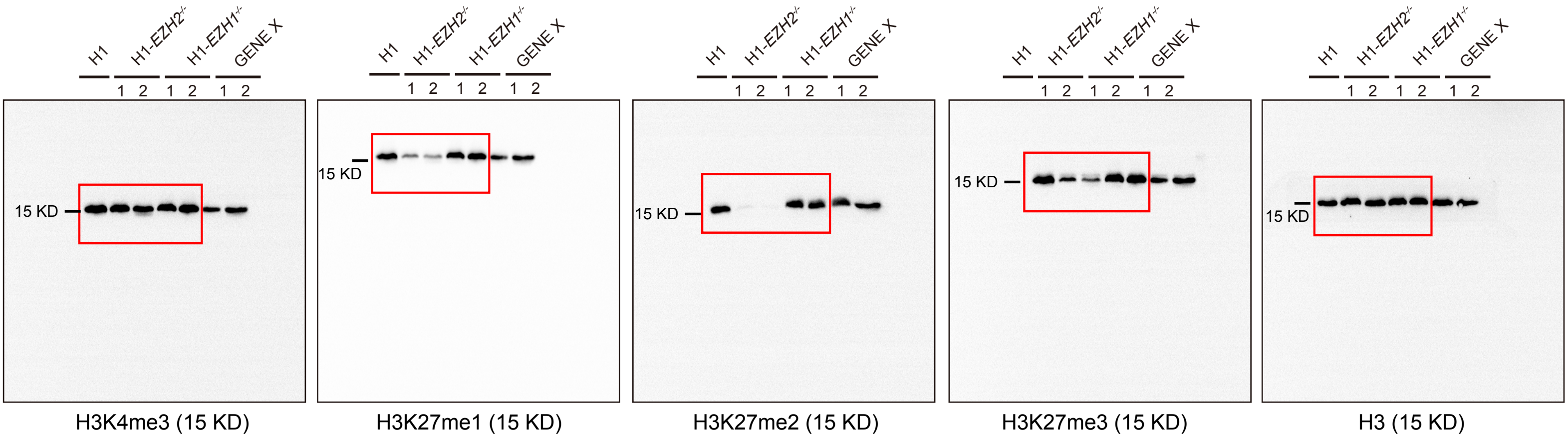

Supplementary Figure 8 uncropped scans of western blots for Figure 1e

(**a**) uncropped scans of western blots for Figure 1e. Histone modifications (H3K4me3, H3K27me1, H3K27me2, H3K27me3) level were analyzed by western-blot using the specific antibody on the whole cell lysates from the indicated cell lines (H1, H1-*EED*$^{-/-}$, H1-*SUZ12*$^{-/-}$ and H1-*EZH2*$^{-/-}$/*EZH1*$^{-/-}$ hESCs). H3 as the loading control. Red box indicates the location of goal protein. KD: Kilodaltons. (**b**) uncropped scans of western blots for Figure 1e. Histone modifications (H3K4me3, H3K27me1, H3K27me2, H3K27me3) level were analyzed by western-blot using the specific antibody on the whole cell lysates from the indicated cell lines (H1, H1-*EZH2*$^{-/-}$, and H1-*EZH1*$^{-/-}$ hESCs). H3 as the loading control. Red box indicates the location of goal protein. KD: Kilodaltons.

Supplementary Figure 9

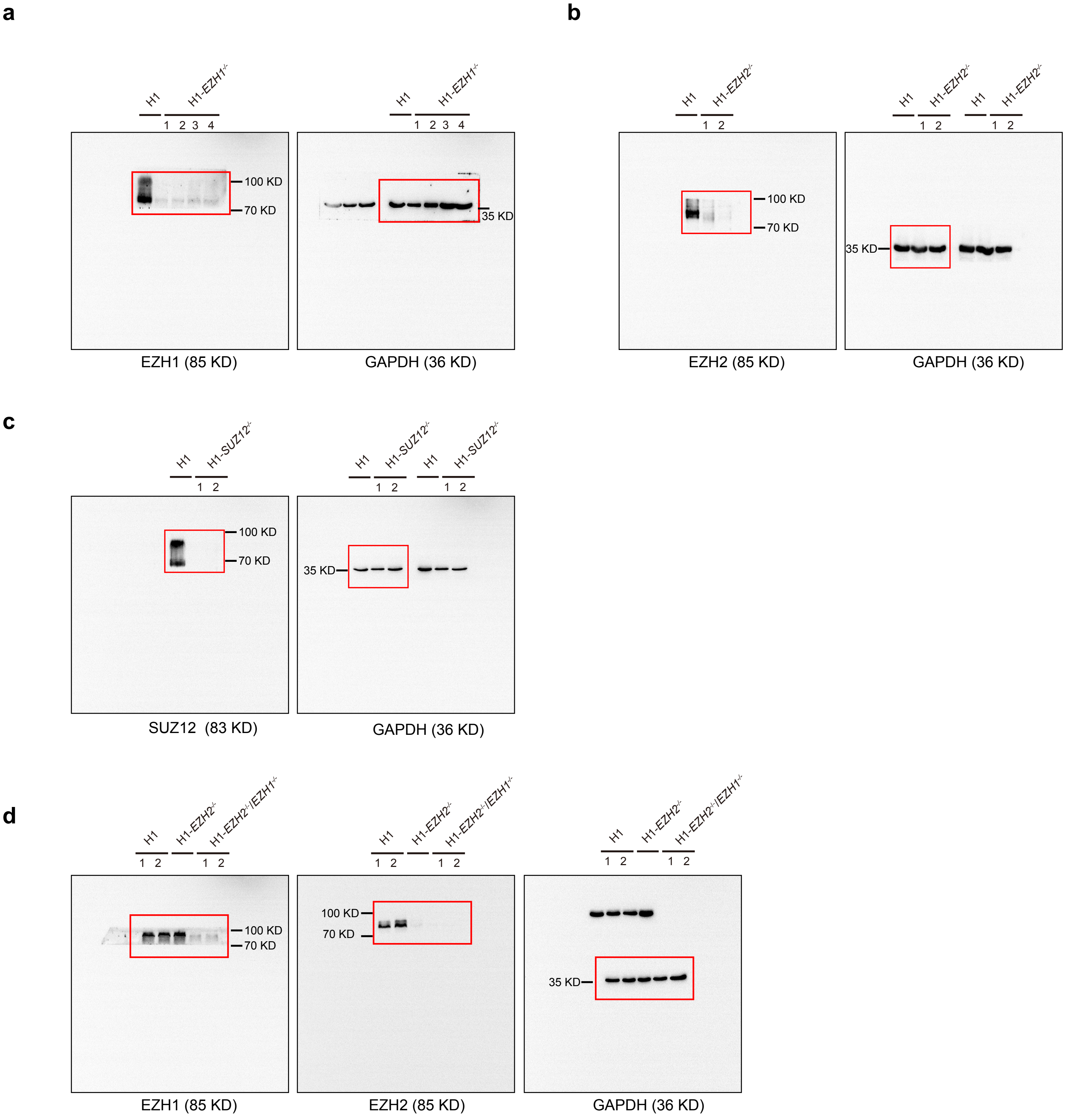

Supplementary Figure 9 uncropped scans of western blots for Supplementary Figure 1b

(**a**) uncropped scans of western blots for Supplementary Figure 1b. EZH1 expression level was analyzed in each indicated cell line (H1, H1-*EZH1*$^{-/-}$ hESCs). GAPDH as the loading control. Red box indicates the location of goal protein. KD: Kilodaltons. (**b**) uncropped scans of western blots for Supplementary Figure 1b. EZH2 expression level was analyzed in each indicated cell line (H1, H1-*EZH2*$^{-/-}$ hESCs). GAPDH as the loading control. Red box indicates the location of goal protein. KD: Kilodaltons. (**c**) uncropped scans of western blots for Supplementary Figure 1b. SUZ12 expression level was analyzed in each indicated cell line (H1, H1-*SUZ12*$^{-/-}$ hESCs). GAPDH as the loading control. Red box indicates the location of goal protein. KD: Kilodaltons. (**D**) uncropped scans of western blots for Supplementary Figure 1b. EZH1 and EZH2 expression level was analyzed in each indicated cell line (H1, H1-*EZH2*$^{-/-}$/*EZH1*$^{-/-}$ hESCs). GAPDH as the loading control. Red box indicates the location of goal protein. KD: Kilodaltons.

Supplementary Table 1 List of sgRNAs and primers for gene targeting

Gene	sgRNA sequence	Primers for validation		Primers for donor DNA construction	
EZH1 (human)	GGGGACCAAGACGTGCAAGC	*EZH1*-F1	CATTCTGCACGCTTCAAAAGCG	*EZH1*-5’ arm-F	AGTGAGCAGGCCTCTGCACTCC
		EZH1-R1	AGAACCTGTGATCAGGGAAATAGGTATG	*EZH1*-5’ arm-R	AGAAGTTGTTGAAGTAGGTGCCATGG
		EZH1-F2	GTTTGTGACAAACTACTGATGACAACTGC	*EZH1*-3’ arm-F	AGACGTGCAAGCAGGTACCATCTG
		EZH1-R2	ACCCAGAAGCATTAAATAGAGTCCAGAC	*EZH1*-3’ arm-R	AGGCCACAGAGTCTCCTCCAGAAG
EZH2 (human)	ATTGCTGGCACCATCTGACG	*EZH2*-F1	CATTCTGCACGCTTCAAAAGCG	*EZH2*-5’ arm-F	AAAGGAGCAGGAGATCAAATCCTCC
		EZH2-R1	GTTGCAAATGCAGTGCTCCTCC	*EZH2*-5’ arm-R	TGCCAGCAATAGATGCTAGAGAATAAAAC
		EZH2-F2	AAAGTGCAGCAGGCCTAGAGCAC	*EZH2*-3’ arm-F	CTCAGAATACTGTGGAGAGGTAAGGCAC
		EZH2-R2	CAATGGGGTCTTGCCATGTTG	*EZH2*-3’ arm-R	CCCTCGTTTCTGAACACTCGGC
EED (human)	ATGGCTCGTATTGCTATCAT	*EED*-F1	CATTCTGCACGCTTCAAAAGCG	*EED*-5’ arm-F	GGGGTTTTGCTATGTTGCCTAGG
		EED-R1	GGGTGGGAGGAGTAAGAAGATCAGG	*EED*-5’ arm-R	GCACAAGTGTAAAAGTTTTCATCAGCC
		EED-F2	AACTACAGCGTGCAGGTTGGTCAC	*EED*-3’ arm-F	TGGCTGTAGCTGGATCTAGAGGCA
		EED-R2	GTCCCTTTGATAACACCCCTCCAG	*EED*-3’ arm-R	TCAGGATGGAGAGGCACTTACTGC
SUZ12 (human)	TATGGAAATACAGACGATTG	*SUZ12*-F1	CATTCTGCACGCTTCAAAAGCG	*SUZ12*-5’ arm-F	ACAAATTTCTGTATTTGATAGATTGCCG
		SUZ12-R1	AAGCATGGGAGAAACACTACTACGAGAG	*SUZ12*-5’ arm-R	GCTTGCTTTTGTTCGTTTTGGC
		SUZ12-F2	AAGAAAATAACTAGTGGGGGTGTGCC	*SUZ12*-3’ arm-F	GAAGATGAAAAGGATCCTGAATGGC
		SUZ12-R2	TTTCACCATGCTGTCCAGACTGG	*SUZ12*-3’ arm-R	CGATGACTCACATCTGTGCAACAAG
Suz12 (mouse)	TCCATTTCTTGTGGCCGAAG	*Suz12*-F1	CATTCTGCACGCTTCAAAAGCG	*Suz12*-5’ arm-F	AACCCAACCAAACCTCACTGCTC
		Suz12-R1	CCCTTAAATCTTTGCAAGGCACC	*Suz12*-5’ arm-R	TCAGATTCAAGAAACTCCGACATGC
		Suz12-F2	TTCTTGAGGGTTCAGAGAGGGAGG	*Suz12*-3’ arm-F	TCCAGAATGGCTGAGAGAAAAAACC
		Suz12-R2	AAAACTTGTATGGGGGCTGGTGAG	*Suz12*-3’ arm-R	AAGCTGAGACCGGCAAATCTTTATG
Eed (mouse)	GCGTATTTGTGGGCGTGTCC	*Eed*-F1	ACTTCATTCCTTGGTTCTTGGTTGG	*Eed*-5’ arm-F	AACTTAGACCACAAAAGCCGTGGG
		Eed-R1	ACCGGTGGATGTGGAATGTGTG	*Eed*-5’ arm-R	GCCACTCTCAATACTGACAGCATCG
		Eed-F2	AGTAATAACACTCTCGCCCCAAATG	*Eed*-3’ arm-F	CAGCCTCAAGGTATGTGCTGGAAG
		Eed-R2	GTGTTGGCATTTGCTAGGGACG	*Eed*-3’ arm-R	GCAGAGGCAGGGAGATCTCTGTATG

Supplementary Table 2 List of primers for over-expression, knockdown and qRT-PCR

Gene	Species	Genebank accession number		Forward primer	Reward primer
EZH1	human		Over-expression	ATGGAAATACCAAATCCCCCTACCTC	AAGGACGTCGGTCTCCCTCTCG
EZH2	human		Over-expression	ATGGGCCAGACTGGGAAGAAATC	AGGGATTTCCATTTCTCTTTCGATG
EED	human		Over-expression	ATGTCCGAGAGGGAAGTGTCGACTG	TCGAAGTCGATCCCAGCGCC
SUZ12	human		Over-expression	ATGGCGCCTCAGAAGCACGG	GAGTTTTTGTTTTTTGCTCTG
EZH1	human		Knockdown	GCTACTCGGAAAGGAAACAAA	TTTGTTTCCTTTCCGAGTAGC
GAPDH	human	NM_014364	qRT-PCR	TCCAAAATCAAGTGGGGCGAT	TTCTAGACGGCAGGTCAGGTC
EZH1	human	NM_001991	qRT-PCR	AATAGCCAGGCTTCTGGGGACC	CAGCTGAATCTTCCTGCAGTGTGC
EZH2	human	NM_015355	qRT-PCR	GCAGGCTGGGGGATTTTTATCA	AACGAATTTTGTTACCCTTGCGG
SUZ12	human	NM_004456	qRT-PCR	TTGCTTTTAGTCGCAACGGACC	TTGTGGACGGAGAGGTAAGCAGG
EED	human	NM_003797	qRT-PCR	TGTTGCAATCTTACGTGGATGCTG	TGATAGCATTTCCATGGCCAACA
OCT4	human	NM_002701	qRT-PCR	CCTCACTTCACTGCACTGTA	CAGGTTTTCTTTCCCTAGCT
SOX2	human	NM_181701	qRT-PCR	CCCAGCAGACTTCACATGT	CCTCCCATTTCCCTCGTTTT
NANOG	human	NM_024865	qRT-PCR	TGAACCTCAGCTACAAACAG	TGGTGGTAGGAAGAGTAAAG
PAX6	human	NM_001604	qRT-PCR	ATGTGTGAGTAAAATTCTGGGCA	GCTTACAACTTCTGGAGTCGCTA
SOX1	human	NM_005686	qRT-PCR	AATTTTATTTTCGGCGTTGC	TGGGCTCTGTCTCTTAAATTTGT
NES	human	NM_145754	qRT-PCR	GAAGGGCAATCACAACAGGTG	GGGGCCACATCATCTTCCA
OTX2	human	NM_172337	qRT-PCR	CAAAGTGAGACCTGCCAAAAAGA	TGGACAAGGGATCTGACAGTG
MIXL1	human	NM_031944	qRT-PCR	GGCGTCAGAGTGGGAAATCC	GGCAGGCAGTTCACATCTACC
T (BRACHYURY)	human	NM_003181	qRT-PCR	TATGAGCCTCGAATCCACATAGT	CCTCGTTCTGATAAGCAGTCAC
RUNX1	human	NM_001754	qRT-PCR	CTGCCCATCGCTTTCAAGGT	GCCGAGTAGTTTTCATCATTGCC
TAL1	human	NM_003189	qRT-PCR	AGCCGGATGCCTTCCCTAT	GGGACCATCAGTAATCTCCATCT
CD31	human	NM_000442	qRT-PCR	AACAGTGTTGACATGAAGAGCC	TGTAAAACAGCACGTCATCCTT
CD34	human	NM_001773	qRT-PCR	CTACAACACCTAGTACCCTTGGA	GGTGAACACTGTGCTGATTACA
FOXA2	human	NM_153675	qRT-PCR	ACTACCCCGGCTACGGTTC	AGGCCCGTTTTGTTCGTGA
SOX17	human	NM_022454	qRT-PCR	CGCACGGAATTTGAACAGTA	GGATCAGGGACCTGTCACAC
HHEX	human	NM_002729	qRT-PCR	GGCAAACCTCTACTCTGGAGC	GTCGTTGGAGAATCTCACCTG
GSC	human	NM_005315	qRT-PCR	AACGCGGAGAAGTGGAACAAG	CTGTCCGAGTCCAAATCGC
EOMES	human	NM_005442	qRT-PCR	CCGCCACCAAACTGAGATGA	ACATTTTGTTGCCCTGCATGT
TFCP2L1	human	NM_014553	qRT-PCR	CAGCCCGAGCACTACAACC	CTCCCAGCTTCCGATTCTCC
BMP4	human	NM_001202	qRT-PCR	ATGTGGGCTGGAATGACTGGAT	ACAGCATGGAGATGGCACTCAG
BMP2	human	NM_198892	qRT-PCR	ACCCGCTGTCTTCTAGCGT	TTTCAGGCCGAACATGCTGAG
BMP5	human	NM_021073	qRT-PCR	AGCGACACCACAAAGAGTTCA	GCTGATGCTCCTGTAAGACTTGA
BMP6	human	NM_001718	qRT-PCR	GCTGCTGGGTTCTAGTGGG	TTCGTGGTTCCGTAGTCTTCTA
TGFB1	human	NM_015927	qRT-PCR	CAAGGGCTACCATGCCAACTTC	CACGTAGTACACGATGGGCAGC
endo-*NANOG*	human	NM_024865	qRT-PCR	GGCCTTAATGTAATACAGCAGACC	CCAGTGTCCAGACTGAAATTGAGT
Gapdh	mouse	NM_008085	qRT-PCR	TGAAGCAGGCATCTGAGGG	CGAAGGTGGAAGAGTGGGAG
Suz12	mouse	NM_199196	qRT-PCR	GAAGATGGAGAAGTGGAGCAGCAG	CATGCTTCATGACATGGAGGTTCC
Oct4	mouse	NM_013633	qRT-PCR	GAAGCAGAAGAGGATCACCTTG	TTCTTAAGGCTGAGCTGCAAG
Sox2	mouse	NM_153559	qRT-PCR	GCGGAGTGGAAACTTTTGTCC	CGGGAAGCGTGTACTTATCCTT
Tfcp2l1	mouse	NM_023755	qRT-PCR	CAGCCCGAACACTACAACCAG	CAGCCGGATTTCATACGACTG
Dppa3	mouse	NM_139218	qRT-PCR	GACCCAATGAAGGACCCTGAA	GCTTGACACCGGGGTTTAG
Sox1	mouse	NM_011439	qRT-PCR	GTGACATCTGCCCCCATC	GAGGCCAGTCTGGTGTCAG
Nes	mouse	NM_016701	qRT-PCR	TCCCTTAGTCTGGAAGTGGCTA	GGTGTCTGCAAGCGAGAGTT
T (Brachyury)	mouse	NM_013729	qRT-PCR	GCTTCAAGGAGCTAACTAACGAG	CCAGCAAGAAAGAGTACATGGC
Runx1	mouse	NM_009822	qRT-PCR	GCAGGCAACGATGAAAACTACT	GCAACTTGTGGCGGATTTGTA
Sox17	mouse	NM_011441	qRT-PCR	GATGCGGGATACGCCAGTG	CCACCACCTCGCCTTTCAC
Foxa2	mouse	NM_010446	qRT-PCR	CCCTACGCCAACATGAACTCG	GTTCTGCCGGTAGAAAGGGA
Bmp4	mouse	NM_007554	qRT-PCR	TGTGAGGAGTTTCCATCACGA	CAGGAACCATTTCTGCTGGGG
Stella	mouse	NM_139218	qRT-PCR	TTCCGAGCTAGCTTTTGAGG	ACACCGGGGTTTAGGGTTAG
Tgfb1	mouse	NM_009365	qRT-PCR	CTTCAATACGTCAGACATTCGGG	GTAACGCCAGGAATTGTTGCTA
Tgfb2	mouse	NM_009367	qRT-PCR	CTTCGACGTGACAGACGCT	GCAGGGGCAGTGTAAACTTATT
Bmp2	mouse	NM_080708	qRT-PCR	GGGACCCGCTGTCTTCTAGT	TCAACTCAAATTCGCTGAGGAC
Eed	mouse	NM_021876	qRT-PCR	CCCACAAATACGCCAAATGCAC	TCCCACAGTTGCAAACACCAGAG

Supplementary Table 3 List of antibodies used in this study

Name of Antibody	Company (Cat. No.)	Dilution Factor
Rabbit anti-EZH1 / ENX-2 Antibody (C-Terminus)	LSBio (C345232)	1:200
SUZ12 (D39F6) XP® Rabbit mAb	Cell Signaling Technology (5246)	1:1000
Ezh2 (D2C9) XP® Rabbit mAb	Cell Signaling Technology (3737)	1:2000
Rabbit anti-HIST3H3 (H3)	Abclonal (A2348)	1:1000
Rabbit anti-Histone H3K27me1 (mono-methyl Lys27)	GeneTex (GTX54104)	1:500
Rabbit anti-Histone H3K27me2 (di-methyl Lys27)	GeneTex (GTX54105)	1:1000
Rabbit anti-Histone H3K27me3 (tri-methyl Lys27)	GeneTex (GTX54106)	1:1000
Rabbit anti-TriMethyl-Histone H3-K4 Polyclonal	Abclonal (A2357)	1:1000
HRP-conjugated Monoclonal Mouse Anti-GAPDH	KangChen Bio-tech (KC-5G5)	1:1000
Goat anti Rabbit IgG HRP	KangChen Bio-tech (KC-RB-035)	1:4000
mouse anti-OCT-3/4	Santa Cruz Biotechnology (sc-5279)	1:200
mouse anti-SSEA4	Invitrogen (414000)	1:200
mouse anti-Isotype antibody mouse IgG2b	Invitrogen (MG2800)	1:200
Goat Anti-Mouse IgG H&L (Alexa Fluor® 488)	Abcam (ab150113)	1:500
Rabbit anti-OCT-3/4	Cell Signaling Technology (2750S)	1:200
Rabbit anti-Glial Fibrillary Acidic Protein Antibody, clone GA5	Millipore (MAB360)	1:1000
Rabbit anti-CALPONIN	Abcam (AB46794)	1:200
mouse anti-SOX17	R&D System (MAB1924)	1:200
Goat anti-Rabbit IgG (H+L) Cross-Adsorbed Secondary Antibody, Alexa Fluor 488	Thermo Fisher SCIENTIFIC (A-11008)	1:500
Goat anti-Mouse IgG (H+L) Cross-Adsorbed Secondary Antibody, Alexa Fluor 568	Thermo Fisher SCIENTIFIC (A-11004)	1:500
Donkey anti-Goat IgG (H+L) Cross-Adsorbed Secondary Antibody, Alexa Fluor 568	Thermo Fisher SCIENTIFIC (A-11057)	1:500
mouse anti-CD34 antibody conjugated with PerCP-Cy5.5	BD Biosciences (347203)	1:100
